# Fructose-2,6-bisphosphate links glucose to survival and growth

**DOI:** 10.1186/2049-3002-2-S1-O5

**Published:** 2014-05-28

**Authors:** Abdullah Yalcin, Yoannis Imbert-Fernandez, Amy Clem, Julie O’Neal, Sucheta Telang, Brian Clem, Jason Chesney

**Affiliations:** 1 JG Brown Cancer Center, University of Louisville, Louisville, KY, USA; 2 Uludag University, Bursa, Turkey; 3 Biochemistry, University of Louisville, Louisville, KY, USA

## Background

Glucose starvation, as occurs in neoplastic tumors, causes G1 arrest and/or apoptosis. Although the mechanisms for these events are poorly understood, reductions in nucleotide synthesis and ATP are widely believed to restrict progression into the S phase and cause apoptosis, respectively. Recently, a side product of glycolysis and stimulator of PFK-1, fructose 2,6-bisphosphate (F2,6BP), was found to be synthesized in the nucleus of cells by the enzyme PFKFB3 and to stimulate proliferation in part by activating cyclin dependent kinases (Cdk). We hypothesized that glucose deprivation would reduce F2,6BP which in turn would suppress the activity of Cdks and cause G1 arrest and apoptosis.

## Materials and methods

Methods included: (i) Western blot for PFKFB3 expression in the cytoplasm and nuclei of A549 cells; (ii) transfection with PFKFB3 siRNA and examination of F2,6BP, Cdk1 activity, p27 expression, cell cycle and apoptosis; (iii) cell cycle analysis after PFKFB3 over-expression; (iv) measurement of recombinant Cdk1, CDC25A-C and Wee1 activities after exposure to F2,6BP; (v) determination of the requirement of multiple regulators of the cell cycle on the proliferative effect of PFKFB3; and (vi) quantification of the effects of glucose deprivation on F2,6BP and the CDC25B substrate phospho-Y15-Cdk1.

## Results

In unpublished results, we demonstrate that: (i) PFKFB3 is selectively expressed in the nucleus during the regulatory G2, M and G1 phases of the cell cycle and in the cytoplasm during the S phase (when glycolysis and DNA synthesis occur); (ii) selection inhibition of PFKFB3 reduces F2,6BP which in turn suppresses Cdk1 activity, increases the Cdk inhibitor p27 and causes a G1/S block and apoptosis; (iii) over-expression of PFKFB3 accelerates G1/S transition; (iv) F2,6BP directly stimulates the activity of recombinant CDC25B which dephosphorylates Y15 in the ATP binding domain of Cdk1 causing activation; (v) selective inhibition of CDC25B or Cdk1 reverses the proliferative effect caused by over-expression of nuclear PFKFB3; and (vi) glucose deprivation reduces F2,6BP and CDC25 activity within 1 hour as determined by increased phospho-Y15-Cdk1 (the dephosphorylation site of CDC25B).

## Conclusions

We conclude that F2,6BP acts as a glucose sensor that regulates survival and cell cycle progression by activating CDC25B during the G1 phase and glucose metabolism by activating PFK-1 during the S phase of the cell cycle. A novel PFKFB3 inhibitor, PFK158, has entered phase I trials and we anticipate that these data will help to develop rational combinations of PFKFB3 inhibitors with other targeted therapies for testing in phase I/II trials.

